# Translating Population Difference: The Use and Re-Use of Genetic Ancestry in Brazilian Cancer Genetics

**DOI:** 10.1080/01459740.2015.1091818

**Published:** 2015-10-09

**Authors:** Sahra Gibbon

**Affiliations:** ^a^ Anthropology Department, University College London, London, England

**Keywords:** Brazil, genetic ancestry, race, risk, South America

## Abstract

In the past ten years, there has been an expansion of scientific interest in population genetics linked to both understanding histories of human migration and the way that population difference and diversity may account for and/or be implicated in health and disease. In this article, I examine how particular aspects of a globalizing research agenda related to population differences and genetic ancestry are taken up in locally variant ways in the nascent field of Brazilian cancer genetics. Drawing on a broad range of ethnographic data from clinical and nonclinical contexts in the south of Brazil, I examine the ambiguities that attention to genetic ancestry generates, so revealing the disjunctured and diverse ways a global research agenda increasingly orientated to questions of population difference and genetic ancestry is being used and reused.

When Paul Rabinow tentatively suggested in the early 1990s that developments in the field of genomics might lead to not just novel configurations of identity but a re-emphasis and invigoration of ‘older’ cultural categories of difference (1996), it was impossible to predict the way that one particular category ‘race’ would take on renewed political and cultural valence in the wake of the genetic and postgenomic research. Despite the announcements that pronounced the so called death of race following discoveries linked to the Human Genome Project in the early 2000s, numerous social scientists have pointed to the powerful resurgence of biological definitions of race in biomedicine and genetic research (Fullwiley 2007; Kahn 2013; Whitmarsh and Jones 2010).

In this article, I contribute to an emerging body of anthropological research exploring how knowledge and research related to genetic ancestry is translated, taken up, and acted on within the field of cancer genetics in Brazil. I examine how particular aspects of a globalizing genomic research agenda, concerned with articulating and engaging with issues of population differences associated with a transnational field of cancer genetic research, is used and re-used in locally variable ways in the nascent field of Brazilian cancer genetics. Drawing primarily on ethnographic data from fieldwork in public and mixed public/private hospitals across three major urban centers in the southern region of Brazil, I examine how questions of population difference and genetic ancestry are expressed, acted on, and in some cases reconfigured for and by practitioners, patients, and their families.

## Genetic ancestry, race, and population difference

Genetic ancestry has become a locus of activity across a broad field of scientific inquiry where the boundaries between research on medical genetics and population genetics are increasingly being blurred and recalibrated (Koenig, Lee, and Richardson 2008). This is especially visible in the growth of direct-to-consumer genetic testing in the United States (Nelson 2008), but increasingly also elsewhere. This raises important questions about the role that ‘geneticized’ historical narratives of migration or origin have for the ‘naturalization’ or ‘biologization’ of pre-existing identities (Palmie 2007), and how they can play a significant role in their transformation (Nelson 2008). In addition, questions have been raised about how a focus on genetic ancestry is informing new understandings of disease risk and etiology for patients and practitioners, as well as impacting on public health interventions.

For some the search for molecular markers or genetic variants that link population differences at the level of the genome to conditions such as diabetes and cardiovascular disease entail a new ‘molecularization’ of race (Fullwiley 2007), biologizing health disparities at the expense of considering social and culture determinants of disease (Kahn 2013, see also Montoya 2011). Others draw attention to how an older era of typological categorization is being re-configured by a focus on genetic ancestry, where there is an emphasis on the clinical distribution of genetic variants and markers across geographically located populations, rather than discreetly defined racial groups (El Haj 2007). As a result a novel form of what Fujimura and Rajagopalan termed “genome geography” (2011:2) has become central to a wide field of genetic research where variations in disease risk and drug response are thought to relate in significant ways to “biogeographical” ancestry profiles. The fact that these profiles are often mapped onto or interpreted in relation to continental scale populations reveals the slipperiness of these categories. It is a process that Kahn has described in terms of “racial recursion,” where categories intended to “avoid racial classifications” actually “loop back to race as they travel” (2013:34).

Nevertheless, while an interest in genetic ancestry is an increasingly evident component of genomic and postgenomic science the meaning and consequences of a renewed attention to population difference is not necessarily homogenous across national and transnational contexts. The Latin American region provides an important cultural context for examining how questions of race and genetic ancestry are being diversely constituted within genomic research (Wade et al. 2014). Brazil in particular has a unique history of race classification and also a complex experience of race, racism, and more recently multiculturalism that directly informs these developments. This has been marked in the colonial period by practices of ‘whitening’ to initially counter what was seen as the “degenerative” effects of colonization (Schwarcz 2008:61). The early and mid-twentieth century, however, saw this process revalorized as *mestiçagem* or race mixture, directly associated with nation building (Santos, Kent, and Neto 2014). These contradictory dimensions have been compounded in the contemporary era by growing evidence of racism across diverse fields of social and public life in Brazil (Hasenbalg 1996), even as this is shown to operate alongside and often through a certain plasticity in the way that a wide range of linguistic descriptors and categories for race and skin color are put to work in the everyday (Telles 2004; Nogueira 1998). This has made engagements with multiculturalism in the region both complex and challenging (Hasenbalg 2005; Guimarães 1999). Population genetic research has recently become part of these complex debates and developments, particularly in relation to discussions about the politics of multiculturalism and affirmative action in public universities (Santos and Maio 2004). Peter Wade and colleagues (2014) suggested that this confluence of diverse historical and contemporary cultural dynamics in Brazil, and other regions of South America, means that genomic research works to not simply ‘reinscribe’ race in these arenas but also to ‘transform it’ (see also, Santos, Silva, and Gibbon 2014). In the following discussion, I critically engage and extend this analysis in examining how genetic ancestry is being translated and mobilized in one specific domain of medical intervention emerging in Brazil.

## The case of BRCA genes

Cancer genetics is one arena where articulations of genetically defined population differences and ancestry have begun to impact not only understandings of disease etiology but also the parameters of medical research and treatment protocols. This has been particularly evident in research and medical interventions linked to the BRCA genes, identified in the mid-1990s and associated with an increased risk of developing breast cancer.

Risk assessment for the BRCA genes, which includes predictive genetic testing for the presence of mutations, has become a routine feature of clinical practice in Euro-American societies, and it is now being variously incorporated across a transnational terrain of research and diverse public/private health institutions. Frequently situated as a ‘preventative’ approach, it is also a field of medical intervention which is helping to ensure that an ‘anticipatory habitus’ is becoming a normalized and routine aspect of what is frequently described as ‘personalized medicine’ (Gibbon et al. 2014). While hundreds of different mutations have been identified on the BRCA genes, certain groups are identified as having a particularly high risk of carrying specific mutations on the BRCA genes. Ashkenazi Jewish communities have been notably singled out in this way because of the way ‘founder effects’ have arisen as a result of social histories and migratory patterns leading to a loss of genetic variation (Mozersky 2013). There has in addition been a growing interest in identifying other ‘founder mutations’ on the BRCA genes, which may be common to specific national contexts or geographically delimited groups (Janavicius 2010). At the same time, the expansion of predictive genetic testing of the BRCA genes to other national contexts has revealed the paucity of international databases of known disease-causing mutations. This has generated considerable uncertainty about the risk conferred by so-called variants of unknown significance, particularly when clinical risk models have been based for the most part on high risk self-identified white individuals from North America and Europe (Kurian 2010). A 2011 commentary in *Nature Genetics* described this in terms of the “missing heritability problem,” calling for urgent action in widening genetic testing so that genotype information from “minority populations” could be included to “improve the case for people of mixed race” to ensure that “those most in need must not be the last to receive the benefits of genetic research” (Bustamante, De La Vega, and Burchard 2011:164). In the United States, the limited value of genetic risk assessment models has been directly linked to the issue of health disparities, related to both the incidence of and mortality from breast cancer. Here efforts to widen access to genetic testing for the BRCA genes are associated with expanding “the preventative potential of genetic testing to reduce cancer incidence” (Hall and Olopade 2006:220; see also Joseph 2014). As a result, in some contexts genomics has become “a plausible solution to racial disparities” (Bliss 2011:1019), which “tethers a discourse of racial justice to notions of racial difference” (Lee 2009:176).

The use of BRCA testing in tumor profiling for those with breast cancer has also seen questions of ancestry and population difference take center stage in efforts to develop targeted treatments for breast cancer. As Sandra Lee suggested, race and ethnicity are now seen “as major axes for stratifying drug response” in the context of breast cancer pharmacogenomics, with different types of breast cancer tumors related to hormone receptor status thought to exist at variable frequencies among different populations defined as “European, Asian and African”(2013:160–161). These developments pose new questions about how genetic variation research is, as Lee suggested, “driving an ontological exercise of further reducing complex disease to subtypes” such that “race [as] a tool in capturing the diversity of breast cancer tumors, becomes re-entangled with how the disease is understood” (2013:161). While there is clear potential here for new forms of ‘niche marketing’ by the pharmaceutical industry, a broader politics of health is also implicated. In this context, the necessary involvement and needs of diverse populations are situated as vital components in fulfilling and making equitable the future health promises of genomics and pharmacogenomics (Lee 2013; see also Taussig and Gibbon 2013).

The expanding scale of medical interventions and research, linked to one high profile arena associated with breast cancer genetics, provides the backdrop against which the recent emergence of cancer genetics in Brazil must be examined. Here categories of genetic ancestry and population difference are heterogeneously mobilized and translated.

## The emerging field of *oncogenética* in Brazil

The development of specialist cancer genetic clinics and services in Brazil has emerged in the past ten years, in the wealthier and relatively more economically developed southern part of the country. With extremely high rates of breast and prostate cancer in these regions (equivalent to the population prevalence in the United States), it is a location that not only reflects regional differences in cancer incidence, but also relative differences in wealth and access to health care services. The constitutional right to health has been in place for over 30 years yet public health provision in Brazil is uneven in its allocation and in its use. Seventy-five percent of the population only access health care through the *Sistema Único de Saúde* (SUS), while the rest of the population use variably affordable health plans or *convenios*. While there are differences in quality of provision both within and across public and private health care, the former is often seen as offering ‘substandard’ care (Edmonds 2010).

During the time of my research (2010–2012), private predictive genetic testing for mutations on the two well-known BRCA genes was available in Brazil for those able to pay approximately $2000 dollars in private clinics. More recently, in 2012, testing was made available via private health insurance providers. Nevertheless the scope of genetic testing in the clinics where I carried out research was, and to a large extent continues to be, directly linked to transnational research collaborations between individual scientists and their research teams, particularly in France, Portugal, and the United States. As a result, there is a complex relationship between emerging clinical services focused on promoting a neglected preventative approach to health care through risk based interventions, and research objectives linked to transnational collaborations (Gibbon 2015).

Next, I present three illustrations of the ways that a discourse about genetic ancestry and categories of population difference, associated with an emerging and global terrain of cancer genetic research and medical practices, are diversely used and reused in Brazilian cancer genetics. These findings draw from an analysis of scientific publications and media discourse, excerpts from interviews with scientists and clinical geneticists, participant observation of clinical encounters and scientific conferences, alongside interviews with patients and their families attending cancer genetic clinics. Field work was undertaken over the course of 18 months between 2010 and 2012 in three cancer genetic clinics in different urban locales in southern Brazil, where I worked with and alongside practitioners, patients, and scientists in public and mixed public/private health care settings. I observed more than 60 clinical consultations in cancer genetic clinics and carried out over 40 interviews with scientific and health professionals working in these settings. This included cancer geneticists, biologists, specialist nurses, bioethicists, and mastologists.^1^ Interviews were carried out with 120 patients and their families attending cancer genetics clinics. They were invited to participate on the basis of either having been offered or because they were awaiting the results of a genetic test related to breast cancer or particular rare cancer syndromes. In a small number of cases, it was possible to carry out interviews prior to and following receipt of a genetic test result. Most interviews were carried out in the hospital environment, although not always at the same time or on the same day as the clinical consultation and mostly in a designated room that had not been used for the clinical appointment.

## ‘Ashkenazi’ mutations in Brazil

Attempts to identity the currently ‘unknown’ nature of genetic risk related to breast cancer in Brazil have fueled a perceived need among cancer genetic research and clinical communities to identify and characterize genetic mutations or markers that might be found at higher frequency in or between different regions of the country. This has informed a plethora of Brazilian studies attempting to examine the prevalence of BRCA mutations in the general Brazilian population, estimated in various studies to be between 3.4% and 9% (Gomes et al. 2007; Ewald et al. 2011). As in the broader global terrain of BRCA genetics, the discovery of new and unknown variants pose significant challenges to scientific and clinical understanding and the communication of risk (Gibbon 2013). Nevertheless the identification in Brazil (even at low frequencies) of well-characterized BRCA mutations, such as those associated with the Ashkenazi Jewish population, has and continues to generate significant research interest (da Costa et al. 2008).

Published scientific and medical articles have speculated as to why these mutations have been found in areas of Brazil where people have not identified as Jewish Ashkenazi. In some cases, this has resulted in an explanatory recourse to historical narratives of migration and colonization. This includes discussion that carriers of these mutations in Brazil were Jews who were expelled from Europe and/or forcibly converted to Christianity following the Edict of Expulsion in Spain and Portugal in the sixteenth century (Gomes et al. 2007). It is a hypothesis that reflects a wider international field of BRCA genetic research, which has begun to examine the presence of Ashkenazi mutations in non-Ashkenazi identified ‘Hispanic’ groups, particularly in the United States, identifying such populations as ‘unknowing Jews’ (Weitzel 2005). Other researchers discussing the presence of BRCA mutations in Brazil have referred to the contribution of ‘Central or Eastern European ancestry’ to the genetic background of the Brazilian population (da Costa et al. 2008), associated with the wave of European immigration to the southern region of Brazil at the turn of the twentieth century.

These scientific explanations reproduce common historical narratives of colonization that are also foundational to popular accounts of Brazilian history; that it is a country formed by a mixture of three ancestral populations typically described in terms of African, Indigenous, and European groups (Santos and Maio 2004). At the same time, a notion of regional specificity is also reproduced in these scientific discussions reflecting the assumed distinctiveness of the south of Brazil, which has long been popularly characterized as constituted by larger numbers of European migrants (Oliven 1996).^2^ While there would appear to be little explicit resistance to the use and reuse of these categories of population difference, in identifying these particular BRCA mutations in non-Ashkenazi identified individuals, the Brazilian research illustrates the instability of the very category ‘Ashkenazi mutations’ (see also Hamel et al. 2011). Of relevance too is how this research focus serves to emphasize the distinctiveness of mixed genetic ancestry and consequently becomes a means of underlining the need for expanded BRCA testing in Brazil.

A number of Brazilian studies identifying BRCA mutations, associated with Ashkenazi populations, at variable but ‘significant’ frequencies, initially called for “ genetic testing for common founder mutations … (for) women with breast cancer before the age of 50 or with a family history of breast or ovarian cancer in a first degree relative” (Gomes et al. 2007:352). Yet other researchers have cautioned against this strategy. Ewald and colleagues (2011) discussed the significance of finding ‘Ashkenazi BRCA mutations’ in Brazil as reflecting the ‘mixed’ ancestry of the Brazilian population rather than the risk constituted by European or Ashkenazi ancestry. They point to the distinctiveness of what are described as Brazilian ‘trihybrid ancestries,’ and how screening *only* for identified Ashkenazi founder mutations (without full gene sequencing) would mean “that more than 90% of mutation carriers would remain unidentified” (2011:6). This highlights the need for a “comprehensive public health approach” that can address the current “lack of coverage … of germline mutation testing” (2011:6). In this case, identification of the Ashkenazi mutations in Brazil is used to underline the mixed ancestry of the Brazilian population, which is itself strategically mobilized in calls for widening the provision of cancer genetics as part of a much needed broader pursuit of public health.

In examining the published scientific discourses in Brazil concerning the meaning and significance of what have become known as the BRCA ‘Ashkenazi Jewish mutations,’ it is evident there are a multiplicity of discourses at stake. These would appear to emphasize colonial histories of migration and regional specificity. Yet the identification of these mutations in Brazil also serves to illustrate population diversity and the limited applicability of a focus only on identified Ashkenazi mutations within the context of the potential public health provision of cancer genetic services.^3^

In the expanding terrain of *Oncogenética* in Brazil, there has been significant interest in another mutation, known as R337h. This has been described in published literature and clinical discourse as a ‘Brazilian founder mutation’ illustrating how the significance of genetic ancestry is being diversely constituted as a means of understanding and addressing cancer etiology in Brazil.

## Between ‘Caucasian haplotypes’ and national identity

Over the past ten years, a germline mutation R337h located on the TP53 gene has been linked to an unusually high incidence of a range of childhood and adult cancers in the southern region of the country. This has been associated with a rare cancer syndrome known as Li-Fraumeni (LFS), whose carriers are estimated to have up to a 90% lifetime chance of developing a range of different cancers. Germline mutations on the TP53 gene are infrequent (1 in 5000 in the United States), yet studies in Brazil indicate that R337h is associated with a high incidence of adrenocortical cancers in children in the state of Paraná (Ribeiro et al. 2001). The decision by the state of Paraná in 2006 to screen all newly born children for R337h has confirmed its high population presence—1 in 300 of all children screened (0.3% of the population). Since 2007, other studies have associated the mutation with breast and other types of cancer in the neighboring southern states of Rio Grande do Sul and Sao Paulo (Achatz Waddington et al. 2007). This has led to the publications on the significance of the mutation for cancer incidence and mortality in the region, with calls for more effective cancer screening and prevention programs “if such a high prevalence of LFS syndrome is confirmed among families in this geographic region” (Palmero et al. 2009:452).

Scientific interest and speculation about the ‘origins’ of the mutation have included efforts to explain its prevalence and seeming regional specificity. This was particularly evident following the publication of a scientific article, which provided what was described as “detailed haplotype analysis” of the R337h mutation, suggesting that “the most likely scenario is that R337h arose in an individual of recent European ancestry” and that the common locus on the gene is associated with a “Caucasian haplotype” (Garritano et al. 2010:146). This theme has also been pursued in various ways in the Brazilian popular press, where more specific historical details have been used to situate this ‘discovery’ within popular narratives of Brazilian colonization and migration.

In an article published in 2011 in the popular national journal *ISTOE*, the plight of families identified as carriers of the mutation was highlighted, while the author also pointed out “that regions in the south and east of the country have the biggest concentration in the world of persons who have an up to 90 percent chance of developing tumors” (Tarantino 2011:72). The author posited that the explanation for the high regional incidence was associated with “a genetic mutation … spread by a Portuguese *Tropeiro* who travelled the region in the south and east in the eighteenth century” (Tarantino 2011:73). The *Tropeiros* are well-known popularized historical figures within Brazil, linked to the arrival of the Portuguese in the seventeenth and eighteenth century, and particularly associated with trading routes that linked the cities on the coast to the mining centers in the mountainous regions that were central to transporting gold and other goods (Pasin 2006). In another article on this discovery in the national press (*Folha de Sao Paulo*) in 2009, one of the clinicians leading the research documented how she had made this association:

We had just arrived in one of the rural towns of Sao Paulo where 85 people from the same family wanted to talk to us. I was in the kitchen of one of the women there when one of them said that her great grandfather had been a *Tropeiro*. This made me think about putting all these families on a map. (Lopes 2009)

Maps illustrating the parallels between the location of those identified with the mutation and the historically documented route of the *Tropeiro* were included in both these discussions in the popular press. The *ISTOE* article concludes with a quote from one of the scientists stating that “we found a unique European common ancestor for everyone” (Tarantino 2011:75).

The emphasis on European ancestry in discussions within scientific reports and published popular media accounts reporting on the R337h mutation might appear to reproduce a globalizing agenda for genomic research related to potentially ‘racialized’ categories of population difference. Nevertheless, while many of the clinicians and scientists in interviews and personal discussions recognized the appeal of the popular historical narrative of the *Tropeiro*, the relevance of specifically Portuguese or European ancestry to an explanation for the high prevalence of R337h in the southern regions of the country was not always explicitly foregrounded. One scientist from Sao Paulo, who worked with the clinical team involved in the research on R337h, explained:

I don’t think of it in terms of ethnicity (*etnia*) it could be I suppose but I’ve never thought about it in this way. It was a person who because of his lifestyle passed this disease to other people and consequently to other generations. … I haven’t picked up on this fact of being Portuguese.

In the clinical consultations I observed, it was rare for the figure of the *Tropeiro* to be mentioned by health professionals; they also did not routinely mention European ancestry or reference to skin color in explanations about cancer risk. While reference was more commonly made to the frequency of the mutation in ‘the south,’ it was not explicitly ‘racialized’ as it seems to have been in some published scientific articles or in the popular Brazilian press. However, a small handful of patients had heard and read the reports in the press, and did occasionally mention these aspects in their consultations or in their discussions with me. For these patients the historical associations they had heard about, linked to the figure of the *Tropeiro*, were more often accompanied by wry smiles and even amusement, rather than any explicit recognition of European ancestry. For instance, one female middle aged patient who had heard of the association with the *Tropeiro* through a television report talked of how she would now ‘joke’ about how “this disgraceful travelling salesman [c*aixeiro viajante*] had to come here!” Another male patient said that he thought the doctor had mentioned this only somewhat ironically, to “lighten the atmosphere” [*quebra a clima*] in the consultation. Commenting on the response of patients, one doctor said, “they think it’s amusing that it was a *Tropeiro* because he was a man who had many lovers and left a lot of family … their interest is because of this not because he had European ancestry.”

These remarks suggest the association of the incidence of R337h with the historical figure of Tropeiro on the one hand make the history of colonization explicit, literalized as a historical product of sexual unions between European men and mostly indigenous or African women—a rendering of colonial history widely articulated in popular discourse including in relation to recent well-publicized population genetic studies in Brazil (Pena et al. 2009). This may in part explain the ‘amused’ response of some patients, as they reflect on the genetic legacy of the amorous activities of the *Tropeiro*. But these geneticized histories also foreground nationalized narratives of population mixture which have also been valorized throughout the twentieth century as a constituting element of Brazilian nationhood and identity (Santos et al. 2014). In this regard, it is significant that despite the absence of an explicit discourse of racialized genetic ancestry in the clinic, R337h was often described in this setting by clinicians and scientists as the ‘Brazilian mutation.’ It is a description that not only articulates national genetic patrimony but also generates a productive ambiguity in the clinical setting, illustrating what Shim and colleagues elsewhere have referred to as the “situated meaning and utility” (2014:18) of genetic ancestry.

The movement between different registers of meaning in how the relevance and significance of R337h is being constituted in Brazil is reflected in a further ethnographic illustration. In December 2011 I attended a national network meeting of the Brazilian Hereditary Cancer Network in Rio De Janeiro where more than 100 health professionals and scientists, with international visitors, had gathered to hear about the latest developments and challenges for cancer genetics in Brazil. The discovery and developments surrounding R337h in Brazil were highlighted in a number of key presentations. In one, the ‘R337h epicenter’ was described as being in the south of Brazil, a geographical location later described by one Brazilian speaker in terms of its association with a particular etnia or ethnicity. There was also further reflection in the same presentation of the link to Portuguese ancestry. This time however the focus was not the figure of the *Tropeiro*, but the Treaty of Tordesillas, which divided the lands of South America between Spain and Portugal in the fifteenth century and which was identified in the presentation as the key historical moment in the ‘arrival’ of the mutation in Brazil from Portugal. Yet despite evidence that the mutation had, thus far, only been identified in the southern regions of the country, there was also a significant emphasis on the public health implications of this research. The prevalence of the mutation was described, for example, as associated with “2000–4000 new cases of cancer a year.” A number of significant images were also shown by one of the researchers during her presentation about R337h. Earlier in her talk, slides from a ‘road trip’ were shown, including a photograph depicting a family in the rural interior state of Sao Paulo. A group of Brazilian and international researchers had met this family, alerted by the very large number of cases of cancer identified across one extended family. As a result of investigations by the scientists some members of the family had been identified as carriers of R337h associated with Li-fraumeni syndrome. The photograph depicted 20 or so members of the same extended family, proudly holding their extensive genealogical tree, flanked by the clinicians and international researchers. At the end of her presentation, seemingly mirroring the contemporary photograph of the family, the clinician showed a well-known painting of a domestic rural setting, painted in the 1920s and entitled The Family by the Brazilian artist Tarsila do Amaral.

When I asked the geneticist why she used this image, she said it was “simply a picture of a typical rural Brazilian family.” Nevertheless many of the Brazilian participants at the scientific meeting would have known that the artist was a leading figure in a well-known politicized cultural movement in the 1920s known as ‘Anthropophagy’ that tried to define and create a distinctively Brazilian art movement not by rejecting European influences but by absorbing and transforming them. Part of this work of transformation was a celebration of racial diversity and *mestiçagem* that came to be integral to the constitution of Brazilian nationhood, as reflected in the typical *mestica* figures in the painting of The Family. It is also significant that the paintings of Tarsila do Amaral have been used by other Brazilian geneticists to explicitly emphasize the importance of rejecting racialized genetic categories of population difference and to instead focus on the unique ‘admixture’ of the Brazilian population (Santos, Silva, and Gibbon 2014). Although accounted for simply in terms of its aesthetic representation as a ‘typical’ rural Brazilian family, the presence of this image at a cancer genetics meeting of both international and national researchers might also be understood in the light of a broader set of cultural meanings. In the same way that the Anthropophagic Movement aimed to incorporate and transform European influences to create a distinctive national art movement, the decision to use this image at the scientific conference reflects an effort to articulate the distinctiveness and relevance of *Brazilian* cancer genetics, a goal in part achieved through the visual emphasis on the relevance of Brazilian *mestiçagem* or mixed genetic ancestries.

The case of R337h explored in scientific publications, media discourse, clinical and scientific narratives and practices, and in patient and practitioner responses, reflects how multiple discourses concerning the meaning of genetic ancestry and population difference are mobilized in pursuit of diverse ends. In my final example, I turn to examine how genetic ancestry is hesitantly and partially incorporated into clinical practice and how patients and their families understand the meaning and significance of cancer risk.

## ‘African American ancestry’ in Brazilian clinical cancer genetics

Carrying out research in cancer genetic clinics in Brazil revealed something of a lacuna in clinical discussions of ethnicity and ancestry. While the Brazilian census categories (black, white, brown, yellow, and indigenous) were sometimes noted by practitioners on the forms that they filled in at the start of a consultation (most often without asking the patient directly), this was not a commonplace practice. As Pagano (2014) pointed out in her recent examination of everyday narratives of race and health in public health clinics in Brazil, there is often very little discussion of racial distinction or identification, despite the requirement by public health ministries now to collect such data.

Asked directly how they would describe their etnia, the majority of patients I met would often be confused or hesitate in their response. Some would refer to their skin color, while a handful were affronted by the question, perceiving it to be racist or inappropriate in some way. Others would simply respond that they were Brazilian, referencing the popular narrative of race mixture in Brazil, or in response to my questions about etnia would talk of cultural attributes such as ‘happiness’ and ‘pride’ in being ‘Brazilian.’ As one patient in Sao Paulo explained when I asked how she would describe her etnia: “It’s complicated this question, because I’m satisfied with my ethnicity. My ancestry is pure Brazilian, and what is Brazilian? Well, it’s mixture (*miscigenação)*, a mixture of races (*raças*). I think it’s beautiful, I’m really proud of this mixture of races.”

Knowledge of historical connections to Europe, mostly from people in the southern city of Porto Alegre, did not preclude their strong sense of also feeling *Brasileiro*, a description often used at the clinical interface as an ethnic identifier.^4^ The diversity of responses to my questions about *etnia* reflect in part the malleability of such identifications in Brazil (Telles 2004).

Despite a common vague sense that having ‘mixed’ ancestry provided protection from diseases such as cancer, only a handful of people explicitly associated having European ancestry with an increased risk for cancer. In discussions with patients, the meaning of genetic ancestry was refracted through relationally and temporally constituted embodied risk, humoral notions of generationally connected body selves, and neo-Lamarckian ideas of inheritance. This raises further questions about how a global agenda of population difference related to genomic medicine is transforming and being informed by culturally relevant understandings of bodies, environment, and cancer risk, even as it dynamically interacts with these different local biologies (Lock and Nguyen 2010; see also Gibbon 2013).

In the final part of this article, I draw on one particular ethnographic moment to illuminate how notions of genetic ancestry are variously silenced, incorporated, and re-configured in the clinical domain of Brazilian cancer genetics, in the negotiations that are undertaken by practitioners and patients to both resist yet also address new and sometimes disorientating information. This makes visible the multiple registers deployed to bridge the gap between a global narrative of risk associated with genetic ancestry and what it means to be *Brasileira* as this relates to diverse and dynamic expressions of population mixture, regional specificity, and also, in this case, skin color and family history.

I had met Claudia, who was in her late fifties, in the cancer genetic clinic in the public hospital in Porto Alegre. She had received a preliminary test result suggesting that she was a likely carrier of a mutation on the BRCA1 gene, and she was waiting for this to be confirmed. When I met her a week later, she told me that the test result had confirmed for her that the cancer was ‘genetic,’ as she had suspected all along. When we talked about her *etnia* or ancestry, she mentioned how, as she put it, “they all came from Germany,” adding “it’s the ‘clear skin’ (*pele clara*).” Although it wasn’t something she had thought a great deal about, she did think that it was her ancestry which she said had “something to do with this [the cancer], with the family from there, that part of Europe, that’s where my ancestors are from.”

Prior to Claudia’s consultation where her result was confirmed, the geneticist had already told me that the mutation identified on the BRCA genes was described in the research literature as being associated with ‘African American ancestry.’ It had been discovered in Claudia’s DNA after a junior member of her team had collaborated with researchers in Portugal. The identification of the mutation was of significant interest and there was a great deal of excitement about having linked this particular mutation to a patient in Brazil, a discovery that was also continuing to generate new international collaborations for the research team. Yet there was also uncertainty for the health professionals involved about the relevance and meaning of the category of ‘African American’ in Brazil. This seemed to be reflected in the response the geneticist made to my query about whether this association was something that would be explained to Claudia. The doctor was surprised by my question, asking why this would be necessary, adding that “they’re all German too” [referring to Claudia’s family] and implying that such news would perhaps not be welcome.

Claudia and her daughter Leane, who was in her mid-twenties, made the next clinical consultation, about a month later, together. I accompanied them and witnessed the same geneticist provide a detailed explanation of the mutation. She outlined why it had been a difficult test to perform, and provided the good news that Leane did not carry the mutation her mother had. Despite this, there was much discussion of the “preventative” screening that each should be engaged in, and the possibility of offering other family members the “opportunity” to be tested. Despite receiving a negative genetic test, Leane was full of questions about appropriate screening for herself. Throughout this half hour conversation a piece of paper lay on the desk in front of them, which both women glanced at periodically. While they had been given a report of their test results in sealed envelopes, the paper in front of them provided a detailed descriptive and a visual representation of the result. The ‘deletion’ was clearly marked with a thick black arrow highlighting the point on the graphic representation of a DNA sequence where it had been found. The Portuguese text above described the mutation in scientific terms, but in English was also written “founder mutation in Afro Americans.” Hardly any reference was made to the paper, although Leane asked to take it away with her at the end of the meeting.

I met with Claudia and Leane half an hour later in a different part of the hospital to talk about the consultation. They were greatly relieved, especially Claudia, who talked about how happy she was to know that it was not “her fault.” Leane was also relieved, but seemed more caught up with the details of the information she had been given. Talking about the impact the information would have for the family, they both spoke about the need to “pay attention … so that people and the family are prepared for this.” Claudia added “it works as prevention—the test would have worked well for her even if it was positive. It is preventative care.” I then asked them directly if they remembered our previous discussion we had had about ancestry, and if they had seen what had been written on the paper that Leane had picked up. Both responded enthusiastically, saying how this had been “interesting,” with Leane pointing out that she had asked the doctor “if it was from Germany, if it was common there” and how the doctor had told her that “the mutation is African, it’s more common in African Americans.” ^5^ There then followed a rapid discussion between the two women as I asked them what they thought about this new piece of information related to ancestry:

Leane: But our origins are all from there. They’re all from Germany.

Claudia: No, but then there is my father’s side. My father’s grandfather was from Germany, they called him *negro Valentim* [Black Valentim] because of the dark skin he had. It was my father’s side, it wasn’t my mother’s side.

Leane: So there you go. There’s someone right there! … but there is so much we don’t know. If we screened everyone, we’d find that everyone is originally from Asia right?

Sahra: How do you think the rest of the family will react to this information about African ancestry?

Leane: No, they are ok with this. Because I hope when you do population genetic screening you see that this shows you are 80% European, right. So then I could be white, but have the genes, you know, like in my case. It’s very interesting; we will tell the family, it’ll be interesting.

Claudia: I think it will be a new thing.

Leane: It will be when we speak to grandma. [Laughter from both women] … it’s all German. They’re all are German. What is not German, they think, “oh Brazilian.” Anyone who doesn’t have a German surname, “ah, he married a Brazilian” … before it was very much like this. … Germans only marry German. Now it’s not like that.

Claudia: Now it is not. But I found it a very interesting subject.

Leane: Interesting because I don’t have it, right!

While initially silenced by the practitioners, the information that the identified mutation on one of Claudia’s BRCA genes was associated with an African ancestry was subsequently partially incorporated into the consultation. As the ethnographic vignettes and exchanges illustrate, it is contextualized by patients (and practitioners) in ways that reveal the different registers through which genetic ancestry is constituted as meaningful in Brazil. The research excitement this finding generates stems as much from the novelty of the identification, the scope of further transnational collaboration and the possibility of more accurately defining the currently ‘unknown’ parameters of genetic ancestry and cancer in Brazil. Of note is the genetic practitioner’s hesitancy in discussing this with the patient and her daughter, given the identification of the family as having German ancestors. I would suggest that this in part reflects a wider set of clinical concerns about the relevance and meaning of geneticized categories of race and ethnicity, linked to international research protocols that may or may not be relevant to risk calculations in Brazil. At the same time, the decision to visually and somewhat implicitly communicate specific information about the association with genetic ancestry to the patient and her daughter also revealed partial acknowledgement of its potential relevancy. While difficult to fully ascertain, it is also possible that this decision could have been a means to revalorize for this particular, ‘German’ identified family the relevance of ‘mixed’ ancestry for Brazilian patients.^6^ While something of a novelty for Claudia and Leane, this new information about their history and identity was also understood and incorporated in different ways. For an older generation, it appeared to challenge pre-existing notions of regionally constituted family identity entailing a reimagining of family history. But at least for Leane, it was accommodated within a wider popular narrative of what it means to be Brazilian, as a place where mixture dominates, where “you could be white” but only be “80% European.”

## Conclusion

Drawing from ethnographic data in southern Brazil on the emergence of cancer genetics, I have examined how novel and increasingly transnational developments in the life and medical sciences are being translated and recalibrated to local contexts in variably specific ways. As the three examples outlined in this article indicate, this is not a process characterized by a unidirectional globalization of population genetic research agendas that uniformly encompass or reproduce seemingly molecularized categories of race and ethnicity. Nor do they suggest their immediate and outright rejection. Rather, there is simultaneously both hesitant resistance and partial incorporation of notions of genetic ancestry and population difference.

In examining how a global terrain of cancer genetics, increasingly attuned to questions of population difference and the relevance of genetic ancestry to knowing and acting on cancer risk, is being translated at the clinical and research interface in Brazil, we see how meaning is constituted through the differently scaled investments of patients and practitioners. The (re)imagined national histories of migration and colonization evident in the work of identifying Ashkenazi mutations and the origin of the R337h mutation powerfully telescope the past and future. Yet these serve to situate Brazil as constituted by both particular ancestries that speak of regional similarity *and* national heterogeneity. In this way, the focus on European ancestry can in different moments express the specificity of regional identity in attending to the demographic differences in cancer incidence or be a component in the popular understanding of Brazil as characterized through ubiquitous mixture. R337h can be associated with a ‘Caucasian haplotype’ or European ancestry and at the same time its origin is linked through historical narratives of colonization to *mestiçagem* as part of a founding myth of Brazil as originating in ancestral mixture. The hesitancy associated with incorporating information about genetic ancestry in the clinical setting provides a further illustration of these multiple meanings as practitioners and patients negotiate the relevance of identifying a mutation in Brazil associated with ‘African American ancestry.’ Susanne Bauer has described such “estimates of susceptibility” in terms of a “lived simulacrum and statistic of the real at the same time” (2014:213) where, in the absence of an ability to carry out wide scale individual or population genotyping, proxies and surrogate variables are materialized and made to matter.

Examining how genetic ancestry is translated in the context of Brazilian cancer genetics makes evident the simultaneous de-stabilizing yet enabling movement between categories of fixity and fluidity, similarity and difference. As Kent, Santos, and Wade (2014) have noted, this not only reflects the “presence and absence of race” in the South American region but the generative movement between “unity” and “diversity” made particularly evident in the ways that notions of race, population difference, national homogeneity, and regional diversity are deployed across diverse fields of genetic research in Brazil. In this way, they pointed out, engagement with biological categories of race in Brazil does not simply reify race or merely reinscribe it, but instead “engages with the ambiguity of race and the multiple modes of imagining the community which are characteristic of the Brazilian milieu” (2014:738).

The pursuit of cancer genetics in Brazil is not simply about the standardized reproduction of global research agendas related to genomics and genetic ancestry. Aligning Brazilian research agendas to transnational priorities is clearly a component of bringing cancer genetics into existence in Brazil, particularly as questions of population difference are increasingly informing the move toward the stratification of diseases such as breast cancer in local and global health care contexts (Lee 2013). Nevertheless, while histories and narratives of colonization, migration and national identity are translated and put to work in both contesting and making relevant biosocial difference in pursuit of promissory futures, research related to population difference is locally calibrated. The contradictory engagement with categories and classifications of population differences in the case studies presented here suggests that rather than explicit resistance, there is strategic mobilization of different notions of genetic ancestry for diverse ends. As a result, disjunctured engagement is likely to be a continued characteristic of Brazilian cancer genetics and also other broader fields of health care, reflecting the ongoing and not easily resolved tensions in the use and reuse of categories of genetic ancestry.

## Funding

This research was funded by the Wellcome Trust (Grant WT084128MA) as part of a project titled “Admixture, Ancestry and Breast Cancer in Brazil: An Ethnographic Investigation of Population Genetics, Disease Risk and Identity.”

## Notes

1. Mastologists are specialists in breast disease.

2. See Kent and colleagues (2014) for further discussion of the way that the distinctiveness of the southern region of Brazil is constituted through genetic research.

3. See Mozersky and Gibbon (2014) for further discussion.

4. See Oliven (1996) for discussion of how the southern regions of the country have historically been identified as being ‘more European.’

5. This exchange happened when I had to momentarily leave the consultation.

6. See Kent and colleagues for a discussion of how associations between sickle cell and African ancestry have been used by Brazilian patient organizations to “create awareness of mixed origins among participants identified as white” (2014:743).
